# Functional decline in hospitalized older patients with coronavirus disease 2019: a retrospective cohort study

**DOI:** 10.1186/s12877-021-02597-w

**Published:** 2021-11-12

**Authors:** Tomohiro Hosoda, Shota Hamada

**Affiliations:** 1 Department of Infectious Disease, Kawasaki Municipal Kawasaki Hospital, 12-1 Shinkawadori, Kawasaki-ku, Kawasaki, 210-0013 Japan; 2 Research Department, Institute for Health Economics and Policy, Association for Health Economics Research and Social Insurance and Welfare, Tokyo, Japan; 3 Department of Health Services Research, Faculty of Medicine, University of Tsukuba, Tsukuba, Japan; 4 Department of Home Care Medicine, Graduate School of Medicine, The University of Tokyo, Tokyo, Japan

**Keywords:** Activities of daily living, Barthel index, Coronavirus disease 2019, Disability, Functional decline, Hospitalization, Older patients

## Abstract

**Background:**

This study aimed to determine the frequency of functional decline and to identify the factors related to a greater risk of functional decline among hospitalized older patients with coronavirus disease 2019 (COVID-19).

**Methods:**

We reviewed the medical records of patients aged over 65 years who were admitted to a tertiary care hospital for COVID-19 over 1 year from February 2020. We evaluated the proportion of functional decline, which was defined as a decrease in the Barthel Index score from before the onset of COVID-19 to discharge. Multivariable logistic regression analyses were performed to evaluate the associations between the demographic and clinical characteristics of patients at admission and a greater risk of functional decline. Two sensitivity analyses with different inclusion criteria were performed: one in patients without very severe functional decline before the onset of COVID-19 (i.e., limited to those with Barthel Index score ≥ 25), and the other with a composite outcome of functional decline and death at discharge.

**Results:**

The study included 132 patients with COVID-19; of these, 72 (54.5%) developed functional decline. The severity of COVID-19 did not differ between patients with functional decline and those without (*P* = 0.698). Factors associated with a greater risk of functional decline included female sex (adjusted odds ratio [aOR], 3.14; 95% confidence interval [CI], 1.25 to 7.94), Barthel Index score < 100 before the onset of COVID-19 (aOR, 13.73; 95% CI, 3.29 to 57.25), and elevation of plasma D-dimer level on admission (aOR, 3.19; 95% CI, 1.12 to 9.07). The sensitivity analyses yielded similar results to those of the main analysis.

**Conclusions:**

Over half of the older patients who recovered from COVID-19 developed functional decline at discharge from a tertiary care hospital in Japan. Baseline activities of daily living impairment, female sex, and elevated plasma D-dimer levels at admission were associated with a greater risk of functional decline.

**Supplementary Information:**

The online version contains supplementary material available at 10.1186/s12877-021-02597-w.

## Background

Since the first patient with coronavirus disease 2019 (COVID-19) was identified in January 2020 in Japan, a total of > 470,000 patients had a confirmed diagnosis of COVID-19. Among > 9000 patients who died from COVID-19 until the end of March 2021 [1], those aged 60 years or older accounted for over 20% [2]. As the disease has now been better characterized, some effective treatments, such as corticosteroids, have been introduced in clinical practice, resulting in better survival than before [3]. However, it has been highlighted as an important challenge to address long-term diseases and conditions due to COVID-19 after discharge from a hospital [4].

Older patients with COVID-19 are more likely to be hospitalized than younger patients, because advanced age is a major risk factor for poor prognosis, including mortality from COVID-19 [5]. However, older age, cognitive impairment, delirium, and hypoalbuminemia are well-known risk factors for hospitalization-associated disability, which is defined as a decrease in independence with activities of daily living (ADL) due to functional decline [6]. In particular, hospitalization due to pneumonia poses a high risk for hospitalization-associated disability, because prolonged inflammation and hypoxia in respiratory infections can cause delirium and cognitive impairment deterioration [7, 8], Similarly, older patients who were hospitalized for the treatment of COVID-19 were considered to be at high risk for functional decline, but little is known about the changes in ADL dependency following the onset of COVID-19.

Therefore, we aimed to determine the frequency of functional decline at discharge from a hospital and to evaluate the factors associated with functional decline in hospitalized older patients with COVID-19.

## Methods

### Design and study population

This was a retrospective cohort study conducted in a tertiary care hospital (Kawasaki Municipal Kawasaki Hospital, Kawasaki, Japan). Japanese citizen aged 65 years or older with COVID-19 who had hospitalized to the study facility between February 1, 2020 and February 28, 2021 were included. All patients were followed-up until their discharge from the hospital (last follow-up date: April 26, 2021). Among them, survivor patients were classified into two groups according to the presence or absence of functional decline from before the onset of COVID-19 to discharge from the hospital, as defined in details below. We performed one main analysis and two sensitivity analyses. One sensitivity analysis was performed to evaluate functional decline in patients after excluding those with the Barthel Index (BI) score < 25 (i.e., very severe physical dependency). Another sensitivity analysis was performed to evaluate the composite outcome of functional decline and death in survivors and those who died during hospitalization.

### Measures and definitions

As the index of ADL, we evaluated the BI which consists of 10 items: feeding, transferring, grooming, toilet use, bathing, mobility, climbing up and down stairs, dressing, ability to control bowel function, and ability to control bladder function. The BI was scored from 0 (completely dependent) to 100 (independent) points, with higher score indicating greater ADL independence [9]. Information on the BI before the onset of COVID-19 was generally collected by interviewing patients in routine clinical practice. For patients who could not communicate with medical staff, we recorded the BI either based on the written information from primary caregiver or nursing home staff or through direct telephone interview with them as soon as possible after admission (within 3 days from admission in most cases). Physicians or nurses evaluated the BI score at discharge by direct observation. The outcome measure was the development of functional decline at discharge, which was defined as a decrease in the BI score of at least 5 points from before the onset of COVID-19 to that at discharge [8], although the minimal important change in the BI has not been established. The patients were also classified into four categories according to the severity of ADL dependency at discharge: independent (the BI score, 100), mildly dependent (75–95), moderate-to-severely dependent (25–70), and very severely dependent (0–20) [10].

We also evaluated age, sex, route of admission, days from the onset of COVID-19 to admission, presence of dementia, and a recorded diagnosis of other comorbidities (chronic obstructive pulmonary disease, chronic kidney disease, diabetes, hypertension, and cardiovascular diseases), which were listed as risk factors for severe COVID-19 according to the Japanese guidelines [11]. COVID-19 severity at admission and the highest severity during hospitalization for COVID-19 were determined according to the classification published by the US National Institutes of Health [12].

Serum albumin, serum C-reactive protein (CRP), and plasma D-dimer levels were measured upon admission. The Geriatric Nutritional Risk Index (GNRI) was measured to assess nutritional status and nutrition-related risk [13]. In addition to baseline characteristics, we also measured the implementation of in-hospital rehabilitation, generally including rehabilitation to maintain limb muscle strength and/or pulmonary rehabilitation in an isolation room for 20 to 40 min per day according to the hospital’s protocol, length of stay (regardless of the type of bed), and discharge destination (in patients admitted from home only).

### Statistical analyses

Characteristics of the patients were compared according to the presence or absence of functional decline (functional decline and death for one sensitivity analysis) using the Fisher’s exact test for categorical variables and the Mann–Whitney U test for continuous variables.

Logistic regression analyses were performed to evaluate the associations between characteristics of the patients at admission and functional decline. We first selected the variables to be evaluated in the associations with functional decline based on the evidence from previous studies [6] as well as those which were considered clinically relevant from the data available. Subsequently, we selected some variables included in the multivariable analyses, taking into account the statistically significant differences between those with or without functional decline in the univariable analysis. As an exception, we used low albumin level as an indicator of malnutrition, because of frequently missing data for the GNRI. We dichotomized the explanatory variables for possible clinical relevance and simple interpretation, including age group (< 80 and ≥ 80 years [very old]), sex, BI score before the onset of COVID-19 (< 100 [dependent] and 100 [independent]), dementia, serum albumin level (< 3.3 and ≥ 3.3 mg/dL), and plasma D-dimer level (< 1.0 and ≥ 1.0 μg/mL). Both the cut-off values for age and for baseline ADL impairment based on the BI score before the onset of COVID-19, which implies dependence in at least one ADL, were based on a previous study which investigated the risk for new-onset disability in hospitalized older adults [14]. The cut-off values for serum albumin and plasma D-dimer levels were determined according to previous reports that evaluated the prognosis of COVID-19 [15, 16]. These evaluations were repeated in two sensitivity analyses with different inclusion criteria. Additionally, considering the possibility of uncertainty in the cut-off values of the explanatory variables, we also performed an analysis with age, the BI score, serum albumin level, and plasma D-dimer level as continuous/ordinal scales.

All analyses were performed using the SPSS (version 15.0.0, Inc., Chicago, IL, USA), and a *P* value < 0.05 was considered statistically significant. The “forestplot” package in R was used to present the results.

### Ethical considerations

This study was conducted in compliance with the principles of the Declaration of Helsinki and current ethical guidelines. This study was approved by the Institutional Review Board of Kawasaki Municipal Kawasaki Hospital (approval number 2020–28) in accordance with the requirements of the institution. The requirement for written informed consent from the patients was waived because of the retrospective nature of this study. The data were analyzed after anonymization.

## Results

### Characteristics of patients

A total of 171 older patients with COVID-19 were hospitalized during the study period. We excluded five patients (2.9%) who were transferred before recovery from COVID-19. In addition, we excluded patients whose BI score before the onset of COVID-19 were missing (*n* = 3) or 0 (*n* = 8), indicating complete dependence of ADL. Of the remaining 155 patients, 23 died (14.8%) during hospitalization. One hundred and thirty-two survivors were included in the main analysis, with a median age of 78.5 years and 49.2% comprising female patients. Seventy-two of the 132 patients (54.5%) were assigned into the functional decline group (FDG), and the remaining 60 into the non-functional decline group (non-FDG).

Demographic and clinical characteristics of the patients are shown in Table 1. Compared with the patients in the non-FDG, those in the FDG were older, had a higher proportion of female patients, had dementia, were admitted from long-term care hospitals/facilities, had lower serum albumin and higher plasma D-dimer levels on admission, and had lower the BI score before the onset of COVID-19. The severity of COVID-19 at admission and serum CRP level at admission were not higher in the FDG.

**Table 1:** Demographic and clinical characteristics of the study patients

	All (*n* = 132)	Functional decline [FDG] (n = 72)	Non-functional decline [Non-FDG] (*n* = 60)	*P* value
Age (years), median (IQR)	78.5 (72.0–85.0)	82.5 (75.8–88.3)	73.0 (70.0–79.0)	< 0.001
Female, n (%)	65 (49.2)	45 (62.5)	20 (33.3)	0.001
Dementia, n (%)	51 (38.6)	43 (59.7)	8 (13.3)	< 0.001
Comorbidities (≥2)^a^, n (%)	54 (40.9)	31 (43.1)	23 (38.3)	0.599
BI score before COVID-19 onset, median (IQR)	100 (80–100)	87.5 (50–100)	100 (100–100)	< 0.001
100 (independent), n (%)	88 (66.7)	31 (43.1)	57 (95.0)	< 0.001
75–95 (mildly dependent), n (%)	14 (10.6)	13 (18.1)	1 (1.7)	
25–70 (moderate-to-severely dependent), n (%)	19 (14.4)	19 (26.4)	0 (0.0)	
< 25 (very severely dependent), n (%)	11 (8.3)	9 (12.5)	2 (3.3)	
Serum albumin level on admission, median (IQR)^b^	3.4 (3.2–3.7)	3.3 (3.1–3.6)	3.5 (3.2–3.8)	0.053
Serum CRP level on admission, median (IQR)	5.3 (1.2–10.4)	5.2 (1.2–10.7)	5.6 (1.5–9.8)	0.940
Plasma D-dimer level on admission, median (IQR)^c^	1.3 (0.8–2.4)	1.5 (1.0–2.6)	1.0 (0.8–2.2)	0.027
GNRI on admission, median (IQR)^d^	99.3 (93.0–107.1)	97.2 (89.6–103.4)	103.3 (95.2–109.3)	0.005
Severity of COVID-19 on admission				
Asymptomatic, n (%)	9 (6.8)	6 (8.3)	3 (5.0)	0.698
Mild, n (%)	15 (11.4)	7 (9.7)	8 (13.3)	
Moderate, n (%)	14 (10.6)	7 (9.7)	7 (11.7)	
Severe, n (%)	76 (57.6)	40 (55.6)	36 (60.0)	
Critical, n (%)	18 (13.6)	12 (16.7)	6 (10.0)	
Duration from the onset of COVID-19 to admission (days), median (IQR)^e^	4.0 (2.0–7.5)	2.0 (1.0–6.0)	6.0 (3.0–9.0)	< 0.001
Route of admission				
Admission from long-term care hospitals/facilities, n (%)	34 (25.8)	32 (44.4)	2 (3.3)	< 0.001
Admission from home, n (%)	98 (74.2)	40 (55.6)	58 (96.7)	

*COVID-19* coronavirus disease 2019, *CRP* C-reactive protein, *FDG* functional decline group, *GNRI* Geriatric Nutritional Risk Index, *IQR* interquartile range

^a^Chronic respiratory disease, chronic kidney disease, diabetes, hypertension, cardiovascular diseases

^b^Data unavailable in one patient in the FDG

^c^Data unavailable in two patients: one in the FDG and another in the non-FDG

^d^Data unavailable in 18 patients: 13 in the FDG and five in the non-FDG

^e^Data unavailable in eight patients: five in the FDG and three in the non-FDG (mainly patients with asymptomatic COVID-19)

### Factors associated with functional decline

**Fig. 1:**
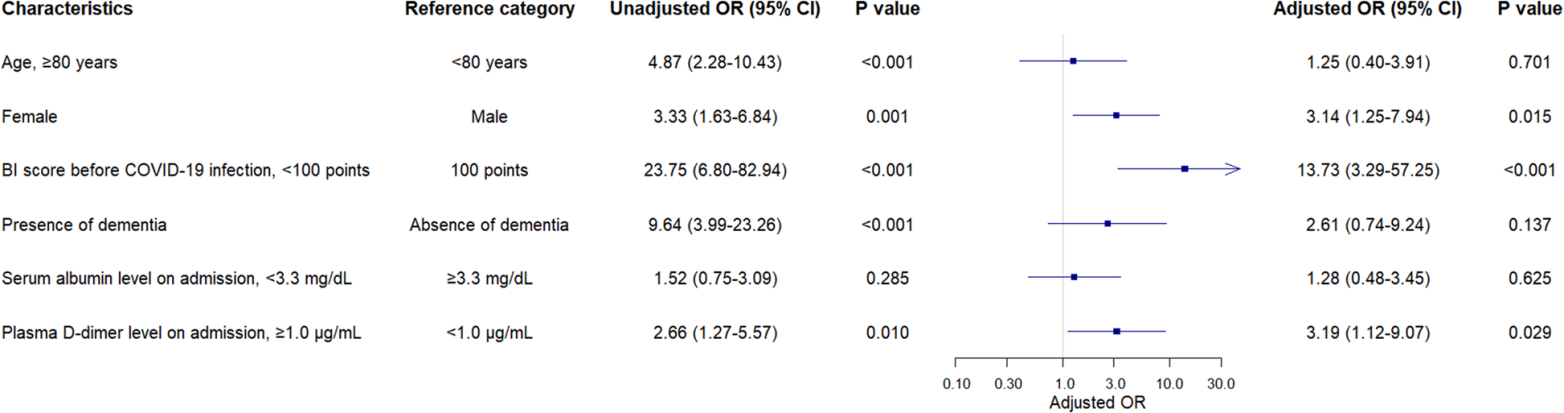
Associations between demographic and clinical characteristics at admission and a greater risk of functional decline. BI, Barthel Index; CI, confidence interval; OR, odds ratio

The associations of baseline characteristics of the patients with a greater risk of functional decline are shown in Fig. 1. In univariable analysis, factors associated with functional decline during hospitalization were age over 80 years (odds ratio [OR], 4.87; 95% confidence interval [CI], 2.28 to 10.43), female sex (OR, 3.33; 95% CI, 1.63 to 6.84), the BI score < 100 before the onset of COVID-19 (OR, 23.75; 95% CI, 6.80 to 82.94), presence of dementia (OR, 9.64; 95% CI, 3.99 to 23.26), and elevation of plasma D-dimer level on admission (OR, 2.66; 95% CI, 1.27 to 5.57).

In multivariable analysis, female sex (adjusted OR, 3.14; 95% CI, 1.25 to 7.94), the BI score < 100 before the onset of COVID-19 (adjusted OR, 13.73; 95% CI, 3.29 to 57.25), and elevation of plasma D-dimer level on admission (adjusted OR, 3.19; 95% CI, 1.12 to 9.07) were independently associated with functional decline.

### Clinical course and outcomes

The clinical course and outcomes are shown in Table 2. The highest severity of COVID-19 during hospitalization was not significantly different between the groups. Most patients in the FDG received rehabilitation services during their hospitalization. The length of stay was longer in the FDG than in the non-FDG. The proportion of patients who were admitted from home but discharged to long-term care hospitals/facilities after recovery from COVID-19 was higher in the FDG than in the non-FDG.

**Table 2:** Clinical course and outcomes

	Functional decline [FDG] (*n* = 72)	Non-functional decline [Non-FDG] (*n* = 60)	*P* value
Highest severity of COVID-19 during hospitalization			
Asymptomatic, n (%)	5 (6.9)	3 (5.0)	0.140
Mild, n (%)	5 (6.9)	7 (11.7)	
Moderate, n (%)	4 (5.6)	7 (11.7)	
Severe, n (%)	38 (52.8)	36 (60.0)	
Critical, n (%)	20 (27.8)	7 (11.7)	
Rehabilitation during the hospitalization, n (%)	59 (81.9)	25 (41.7)	< 0.001
Length of stay (days), median (IQR)	17.0 (12.0–35.3)	11.0 (8.0–15.3)	< 0.001
Admission from home but discharge to long-term care hospitals/facilities, n (% of patients admitted from home)	24 (60.0)	3 (5.2)	< 0.001
***Total BI score***			
BI score at discharge, median (IQR)	27.5 (0–60)	100 (100–100)	
100 (independent), n (%)	0 (0.0)	57 (95.0)	
75–95 (mildly dependent), n (%)	14 (19.4)	1 (1.7)	
25–70 (moderate-to-severely dependent), n (%)	24 (33.3)	0 (0.0)	
< 25 (very severely dependent), n (%)	34 (47.3)	2 (3.3)	
Decline in BI score, median (IQR)	35 (15–50)	–	
Worsened BI category before the onset of COVID-19, n (%)	56 (77.8)	–	
Impairment in each ADL			
Feeding (eating self any normal food), n (%)	38 (53.5)	0 (0.0)	
Transfer (moving from wheelchair to bed and return), n (%)	47 (66.2)	0 (0.0)	
Grooming (personal hygiene), n (%)	25 (35.2)	0 (0.0)	
Toilet use (transferring to and from a toilet), n (%)	39 (54.9)	0 (0.0)	
Bathing (getting in and out, and washing self), n (%)	31 (43.7)	1 (1.4)	
Mobility (walking on a level surface), n (%)	49 (69.0)	0 (0.0)	
Stairs (going up and down stairs), n (%)	37 (52.1)	0 (0.0)	
Dressing (selecting and putting on all clothes), n (%)	45 (63.4)	0 (0.0)	
Bowels (continent), n (%)	43 (60.6)	1 (1.4)	
Bladder (continent), n (%)	39 (54.9)	0 (0.0)	
The number of impaired ADL components, median (IQR)	5 (3–8)	0 (0–0)	

*ADL* activity of daily living, *BI* Barthel Index, *COVID-19* coronavirus disease 2019, *FDG* functional decline group, *IQR* interquartile range

^a^For example, from mildly dependent to moderate-to-severely dependent

Among the 132 study patients, 60 (45.5%) had at least moderate dependence (i.e., the BI score < 75) at discharge. In the FDG (*n* = 72), approximately 80% of the patients were moderate to very severe dependent. In addition, 56 of 72 patients in the FDG (77.8%) developed a worsened category of the severity of ADL dependency (e.g., from mildly dependent to moderate-to-severely dependent).

In the FDG, the median decrease in the BI score was 35 points, and the median number of impaired BI components was five. Walking on a level surfaces (69.0%), moving from a wheel chair to a bed and returning (66.2%), and dressing (63.4%) were often impaired in the FDG.

In the non-FDG, two patients changed the two components of the BI without decreasing the total BI score. One patient had an impaired bathing component from independent to dependent, whereas the bladder component improved from incontinent to occasional accident. The other patient had an impaired bowel component from occasional accident to incontinent, whereas the feeding component improved from unable to need help.

### Sensitivity analyses

The analysis with age and the BI score as continuous/ordinal variables yielded similar results to those of the main analysis: a 10-year increase in age was not associated with a greater risk of functional decline (adjusted OR, 1.04; 95% CI, 0.52 to 2.09), but a 5-point decrease in the BI score was (adjusted OR, 1.16; 95% CI, 1.01 to 1.33). However, the association between dementia and a greater risk of functional decline reached statistical significance (adjusted OR, 4.83; 95% CI, 1.47 to 15.85), whereas a 1 μg/mL increase in plasma D-dimer level was not statistically significantly associated with a greater risk of functional decline (adjusted OR, 1.26; 95% CI, 0.98 to 1.62) (Fig. S1) (Additional file 1).

One sensitivity analysis included 121 patients without very severe ADL dependence before the onset of COVID-19, and of these, 63 (52.1%) developed functional decline. The other sensitivity analysis included 155 survivors and those who died, and 95 patients (61.3%) developed functional decline or death. The differences between the patients with individual outcomes and those without in sensitivity analyses were similar to those in the main analysis, with some exceptions: lower serum albumin level in patients with the outcomes (with statistical significance) and the highest severity of COVID-19 during hospitalization in those with functional decline or death (Tables S1, S2, and S3) (Additional file 1). The sensitivity analyses yielded similar results to those of the main analysis; thus, female sex (not reaching statistically significant difference in the sensitivity analysis including survivors and those who died), the lower BI score before the onset of COVID-19, and elevation of plasma D-dimer level were associated with greater risks for functional decline or functional decline and death (Figs. S2 and S3) (Additional file 1).

## Discussion

From this retrospective cohort study, we showed that the majority of older patients who recovered from COVID-19 developed functional decline during hospitalization, especially in their ability of walking on a level surface and moving from wheelchair to bed and return. One of the strengths of this study was the availability of data on the ADL before the onset of COVID-19, and evaluated the changes in the BI score until discharge from the acute care hospital. In addition, the factors associated with functional decline included female sex, ADL dependency before the onset of COVID-19, and the elevation of plasma D-dimer level on admission.

Our results may be useful in selecting appropriate candidates for rehabilitation during hospitalization from limited rehabilitation resources to treat and prevent functional decline and to provide the high-risk patient families or caregivers with sufficient time to arrange support after the discharge home (e.g., home help services and a reclining bed for easy transferring from a bed). These implications would be supported by a previous study, which showed that multi-domain assessment of COVID-19 inpatients, including functional, physical, and cognitive assessment, may be useful for evaluating their health status and for planning care, rehabilitation, and follow-up after discharge [17].

The proportion of functional decline in our study, defined by decreasing the BI score at discharge from before the onset among the patients who recovered from COVID-19, might be higher than that in a previous study. A multicenter study conducted in Norway with older patients (mean age, 74.3 years) who recovered from COVID-19 has shown that ADL impairment persisted in 35% of patients 6 months after discharge [18]. The proportion of ADL impairment at discharge might be also higher in the present study. A multicenter study conducted in China with a relatively younger COVID-19 patient population (median age, 49 years) has shown that the proportion of at least moderate ADL dependence due to COVID-19 defined by a BI score < 75 at discharge was 16.4% [19]. The diversity in characteristics of patients, including age, the definition of functional decline or ADL impairment, and the duration of follow-up to evaluate the ADL impairment may affect the differences between the results from the previous studies and ours.

In addition, functional decline due to COVID-19 in our study sample was more frequent than that in patients with other diseases which were previously reported. A meta-analysis reported the prevalence of a loss of independence with ADL as 30% (95% CI, 24 to 33%) in acute care hospital units, despite a large heterogeneity between studies [20]. Another study has shown that moderate to catastrophic functional decline following hospitalization for influenza or acute respiratory illness was 18.2% [21]. The difference in the frequency of functional decline between our study and the previous studies may be partly due to stricter isolation to prevent the secondary infection of COVID-19 during hospitalization, which might also disturb adequate rehabilitation to maintain ADL. Moreover, staying in solitude due to restricted family visits may cause the development of functional decline and cognitive impairment [22]. Considering the higher proportion of patients’ ADL impairment at discharge, especially in transferring and mobility in the patients discharged from hospitals, attention should be paid to a potentially increased risk of falls. Family members or caregivers, as well as healthcare providers, should also prepare the support for functional decline, including rehabilitation after discharge [23], for a patient who is being discharged from a hospital.

ADL impairment before the onset of COVID-19, which was identified as a strong factor associated with functional decline, is also known as a risk factor for in-hospital mortality among older patients with COVID-19 [24, 25]. Among patients with pneumonia or acute respiratory infection, older age, cognitive impairment, malnutrition, and baseline ADL impairment are well-known risk factors for hospital-associated disability [21, 26]. Notably, the risk of functional decline at discharge was higher even in patients with mild ADL impairment before the onset of COVID-19 than in those without ADL impairment in our study.

Several previous studies have reported that the elevation of plasma D-dimer level was associated with the clinical frailty scale in older patients [27] and with the development of functional disability through the subclinical activation of coagulation and inflammatory cascades [28]. Our study may also indicate the association between vascular endothelial damage due to excessive inflammation in COVID-19 patients and functional decline. In contrast, a study has shown that the severity of COVID-19 may have little effect on the extent of instrumental ADL limitations in survivors aged over 60 years [19]. According to a recent study, the BI score at discharge after recovery from COVID-19 was not significantly different according to whether the patient stayed in an ICU or not [17]. Therefore, clinicians should note that functional decline could develop in patients with the risk factors identified in this study even without severe COVID-19.

Our study has four major limitations. First, the use and measurement of the BI in the present study has several potential limitations: the ceiling effect, accuracy of recorded data, measurement settings, and follow-up data. The BI is known to be more susceptible to a ceiling effect than the other scales for evaluating ADL [29, 30]. However, even considering the ceiling effect, we should note that 31 of 88 patients whose BI score was 100 at admission (35.2%) experienced functional decline during the hospitalization for COVID-19 treatment. In addition, the BI score before the onset of COVID-19 collected by telephone interviews with family members or caregivers might not be accurate; however, a previous report showed the reliability and validity of telephonic BI [31], and we made an effort to interview family members or primary caregivers as soon as possible after admission. Moreover, we evaluated the BI score at discharge in an isolated hospital room, and the BI score may be influenced by the difference in the measurement settings, such as their familiar home or nursing homes. We did not evaluate the persistence of functional decline after discharge from the hospital. Because of the tertiary care hospital setting in this study, we accepted patients from a large number of hospitals and facilities or living in a wide area. This limits the examination of outpatient follow-up after discharge. However, as described in a previous study [19], functional decline may also persist in at least a part of patients in our study population.

Second, this study was dependent on the accuracy of the recorded data and the range of variables because of its retrospective nature. For example, the diagnosis of dementia may not always be assessed using the same diagnostic test or may not be always recorded. This could introduce a bias at evaluating the association of presence of dementia with development of functional decline. In addition, we could not exclude the possibility of residual confounding, such as delirium [32], in the associations between the factors identified and functional decline.

Third, the cut-off values of some variables (i.e., age and the BI score) might not be sufficiently justified, although the sensitivity analyses generally showed similar results to those of the main analysis except for some differences in statistical significance. Therefore, further studies with larger study samples with different cut-off points or categorization should be conducted to verify the findings from this study.

Fourth, because this was a single-center study in a tertiary care hospital in Japan, where the mortality rate of COVID-19 was lower than that in most Western countries [33], it did not include a wide range of COVID-19 patients. Therefore, the association between the characteristics of the patients and functional decline may be biased, and, the generalizability of the findings from this study may be limited, although the impact of the difference in our study findings cannot be exactly estimated. Further investigations including various types of medical institutions or from various countries or regions with different mortality rates could resolve the limitation.

## Conclusions

Functional decline from before the onset of COVID-19 to discharge was prevalent in older patients admitted to a Japanese tertiary care hospital. Baseline ADL impairment, female sex, and elevated plasma D-dimer level were associated with a greater risk of functional decline.

## Supplementary Information

**Additional file 1: Table S1**. Demographic and clinical characteristics of the study patients without very severe functional impairment before coronavirus disease 2019 onset**. Table S2**. Demographic and clinical characteristics of the study patients, including survivors and those who died. **Table S3**. Clinical courses and outcomes in the study patients including the sensitivity analyses. **Figure S1**. Associations between demographic and clinical characteristics at admission and a greater risk of functional decline in the analysis with age, the BI score, serum albumin level, and plasma D-dimer level as continuous/ordinal scales. **Figure S2**. Associations between the demographic and clinical characteristics at admission and a greater risk of functional decline in patients without very severe functional impairment before coronavirus disease 2019 onset. **Figure S3**. Associations between the demographic and clinical characteristics at admission and a greater risk of functional decline or death in patients, including survivors and those who died.

## Data Availability

The datasets used and analysed during this study are available from the corresponding author on reasonable request after the consultation with the Institutional Review Boards at the study hospital.

## Abbreviations

ADL: Activity of daily living
BI: Barthel Index
CI: Confidence interval
COVID-19: Coronavirus disease 2019
FDG: Functional decline group
GNRI: Geriatric Nutritional Risk Index
OR: Odds ratio
