# Supplementary material for: Functional decline in hospitalized older patients with coronavirus disease 2019: a retrospective cohort study
Source: BMC Geriatr. 2021 Nov 12;21:638. doi: 10.1186/s12877-021-02597-w (PMC8586826; doi:10.1186/s12877-021-02597-w)
Supplement: Supplementary file 1 — Additional file 1: Table S1. Demographic and clinical characteristics of the study patients without very severe functional impairment before coronavirus disease 2019 onset. Table S2. Demographic and clinical characteristics of the study patients, including survivors and those who died. Table S3. Clinical courses and outcomes in the study patients including the sensitivity analyses. Figure S1. Associations between demographic and clinical characteristics at admission and a greater risk of functional decline in the analysis with age, the BI score, serum albumin level, and plasma D-dimer level as continuous/ordinal scales. Figure S2. Associations between the demographic and clinical characteristics at admission and a greater risk of functional decline in patients without very severe functional impairment before coronavirus disease 2019 onset. Figure S3. Associations between the demographic and clinical characteristics at admission and a greater risk of functional decline or death in patients, including survivors and those who died. [file 12877_2021_2597_MOESM1_ESM.pdf]

Additional file 1

Table S1. Demographic and clinical characteristics of the study patients without very severe functional impairment before coronavirus disease 2019 onset

Table S2. Demographic and clinical characteristics of the study patients, including survivors and those who died

Table S3. Clinical courses and outcomes in the study patients including the sensitivity analyses

Figure S1. Associations between demographic and clinical characteristics at admission and a greater risk of functional decline in the analysis with age, the BI score, serum albumin level, and plasma D-dimer level as continuous/ordinal scales

Figure S2. Associations between the demographic and clinical characteristics at admission and a greater risk of functional decline in patients without very severe functional impairment before coronavirus disease 2019 onset

Figure S3. Associations between the demographic and clinical characteristics at admission and a greater risk of functional decline or death in patients, including survivors and those who died

Table S1. Demographic and clinical characteristics of the study patients without very severe functional impairment before coronavirus disease 2019 onset

	All (n=121)	Functional decline [FDG] (n=63)	Non-functional decline [Non-FDG] (n=58)	P value
Age (years), median (IQR)	78.0 (71.0–84.0)	82.0 (75.5–88.5)	73.0 (70.0–79.0)	<0.001
Female, n (%)	60 (49.6)	41 (65.1)	19 (32.8)	0.001
Dementia, n (%)	41 (33.9)	35 (55.6)	6 (10.3)	<0.001
Comorbidities (≥2)[a], n (%)	52 (43.0)	29 (46.0)	23 (39.7)	0.582
BI score before COVID-19 onset, median (IQR)	100 (95–100)	95 (70–100)	100 (100–100)	<0.001
100 (independent), n (%)	88 (72.7)	31 (49.2)	57 (98.3)	<0.001
75–95 (mildly dependent), n (%)	14 (11.6)	13 (20.6)	1 (1.7)	
25–70 (moderately-to-severely dependent), n (%)	19 (15.7)	19 (30.2)	0 (0.0)	
Serum albumin level on admission, median (IQR)	3.4 (3.2–3.7)	3.3 (3.0–3.6)	3.5 (3.2–3.8)	0.030
Serum CRP level on admission, median (IQR)	5.8 (1.2–10.3)	5.8 (1.4–10.9)	5.6 (1.3–9.7)	0.817
Plasma D-dimer level on admission, median (IQR)[b]	1.3 (0.8–2.3)	1.5 (0.9–2.6)	1.0 (0.8–2.2)	0.020
GNRI on admission, median (IQR)[c]	99.5 (93.2–107.1)	97.4 (90.4–102.8)	103.4 (95.7–109.4)	0.004
Severity of COVID-19 on admission				
Asymptomatic, n (%)	7 (5.8)	5 (7.9)	2 (3.5)	0.466
Mild, n (%)	14 (11.6)	6 (9.5)	8 (13.8)	
Moderate, n (%)	13 (10.7)	7 (11.1)	6 (10.3)	
Severe, n (%)	69 (57.0)	33 (52.4)	36 (62.1)	
Critical, n (%)	18 (14.9)	12 (19.0)	6 (10.3)	

Duration from the onset of COVID-19 to admission (days), median (IQR)[d]	4.0 (2.0–7.5)	2.0 (1.0–6.0)	6.0 (3.8–9.0)	<0.001
Route of admission				
Admission from LTC hospitals/facilities, n (%)	24 (19.8)	23 (36.5)	1 (1.7)	<0.001
Admission from home, n (%)	97 (80.2)	40 (63.5)	57 (98.3)	

BI, Barthel Index; COVID-19, coronavirus disease 2019; CRP, C-reactive protein; FDG, functional decline group; GNRI, Geriatric Nutritional Risk Index; IQR, interquartile range; LTC, long-term care

a. Chronic respiratory disease, chronic kidney disease, diabetes, hypertension, and cardiovascular diseases

b. Data unavailable in one patient in the non-FDG

c. Data unavailable in 13 patients; nine in the FDG and four in the non-FDG

d. Data unavailable in six patients: four in the FDG and two in the non-FDG (mainly patients with asymptomatic COVID-19)

Table S2. Demographic and clinical characteristics of the study patients, including survivors and those who died

	All (n=155)	Functional decline or death [FDG] (n=95)	Non-functional decline [Non-FDG] (n=60)	P value
Age (years), median (IQR)	79.0 (72.0–85.5)	83.0 (76.0–88.0)	73.0 (70.0–79.0)	<0.001
Female, n (%)	76 (49.0)	56 (58.9)	20 (33.3)	0.003
Dementia, n (%)	64 (41.3)	56 (58.9)	8 (13.3)	<0.001
Comorbidities (≥2)[a], n (%)	66 (42.6)	43 (45.3)	23 (38.3)	0.410
BI score before COVID-19 onset, median (IQR)	100 (70–100)	85 (50–100)	100 (100–100)	<0.001
100 (independent), n (%)	96 (61.9)	39 (41.1)	57 (95.0)	<0.001
75–95 (mildly dependent), n (%)	19 (12.3)	18 (18.9)	1 (1.7)	
25–70 (moderately-to-severely dependent), n (%)	26 (16.8)	26 (27.3)	0 (0.0)	
<25 (very severely dependent), n (%)	14 (9.0)	12 (12.6)	2 (3.3)	
Serum albumin level on admission, median (IQR)[b]	3.4 (3.2–3.7)	3.3 (3.1–3.6)	3.5 (3.2–3.8)	0.026
Serum CRP level on admission, median (IQR)	5.8 (1.4–10.9)	5.8 (1.4–11.8)	5.6 (1.5–9.8)	0.731
Plasma D-dimer level on admission, median (IQR)[c]	1.4 (0.9–2.5)	1.5 (1.0–2.8)	1.0 (0.8–2.2)	0.005
GNRI on admission, median (IQR)[d]	98.7 (92.4–107.0)	96.9 (88.7–103.1)	103.3 (95.2–109.3)	0.001
Severity of COVID-19 on admission				
Asymptomatic, n (%)	9 (5.8)	6 (6.3)	3 (5.0)	0.510
Mild, n (%)	17 (11.0)	9 (9.5)	8 (13.3)	
Moderate, n (%)	14 (9.0)	7 (7.4)	7 (11.7)	
Severe, n (%)	91 (58.7)	55 (57.9)	36 (60.0)	
Critical, n (%)	24 (15.5)	18 (18.9)	6 (10.0)	

Duration from the onset of COVID-19 to admission (days), median (IQR)[e]	3.0 (2.0–7.0)	2.0 (1.0–5.0)	6.0 (3.0–9.0)	<0.001
Route of admission				
Admission from LTC hospitals/facilities, n (%)	44 (28.4)	42 (44.2)	2 (3.3)	<0.001
Admission from home, n (%)	111 (71.6)	53 (55.8)	58 (96.7)	

BI, Barthel Index; COVID-19, coronavirus disease 2019; CRP, C-reactive protein; FDG, functional decline group; GNRI, Geriatric Nutritional Risk Index; IQR, interquartile range; LTC, long-term care

a. Chronic respiratory disease, chronic kidney disease, diabetes, hypertension, and cardiovascular diseases

b. Data unavailable in one patient in the non-FDG

c. Data unavailable in two patients: one in the FDG and one in the non-FDG

d. Data unavailable in 23 patients: 18 in the FDG and five in the non-FDG

e. Data unavailable in eight patients: five in the FDG and three in the non-FDG (mainly patients with asymptomatic COVID-19)

Table S3. Clinical courses and outcomes in the study patients including the sensitivity analyses

		Functional decline [FDG]	Non-functional decline [Non-FDG]	P value
Study patients without very severe functional impairment before coronavirus disease 2019 onset				
N		63	58	
Highest severity of COVID-19 during hospitalization	Asymptomatic, n (%)	4 (6.3)	2 (3.4)	0.113
	Mild, n (%)	4 (6.3)	7 (12.1)	
	Moderate, n (%)	4 (6.3)	6 (10.3)	
	Severe, n (%)	32 (50.8)	36 (62.1)	
	Critical, n (%)	19 (30.2)	7 (12.1)	
Rehabilitation during the hospitalization, n (%)		31 (49.2)	25 (43.1)	<0.001
Length of stay (days), median (IQR)		17.0 (11.5–35.5)	11.0 (8.0–15.0)	<0.001
Admission from home but discharge to LTC hospitals/facilities, n (% of patients admitted from home)		24 / 40 (60.0)	2 / 57 (3.5)	<0.001
Study patients including survivors and those who died				
N		95	60	
Highest severity of COVID-19 during hospitalization	Asymptomatic, n (%)	5 (5.3)	3 (5.0)	0.001
	Mild, n (%)	6 (6.3)	7 (11.7)	
	Moderate, n (%)	4 (4.2)	7 (11.7)	
	Severe, n (%)	38 (40.0)	36 (60.0)	
	Critical, n (%)	42 (44.2)	7 (11.7)	
Rehabilitation during the hospitalization, n (%)		72 (75.8)	25 (41.7)	<0.001
Length of stay (days), median (IQR)		16.0 (12.0–28.5)	11.0 (8.0–15.3)	<0.001
Admission from home but discharge to LTC hospitals/facilities, n (% of patients admitted from home)		24 / 53 (45.3)	3 / 58 (5.2)	<0.001

COVID-19, coronavirus disease 2019; FDG, functional decline group; IQR, interquartile range; LTC, long-term care

Figure S1. Associations between demographic and clinical characteristics at admission and a greater risk of functional decline in the analysis with age, the BI score, serum albumin level, and plasma D-dimer level as continuous/ordinal scales

Characteristics	Reference	Unadjusted OR (95% CI)	P value	Adjusted OR (95% CI)	P value
Age, 10-year increase	–	2.85 (1.75–4.65)	<0.001	1.04 (0.52–2.09)	0.919
Female	Male	3.33 (1.63–6.84)	0.001	3.95 (1.53–10.20)	0.005
BI, 5-point decrease	–	1.29 (1.13–1.48)	<0.001	1.16 (1.01–1.33)	0.033
Dementia	Absence	9.64 (3.99–23.26)	<0.001	4.83 (1.47–15.85)	0.009
Albumin, 1 mg/dL decrease	–	1.95 (0.96–3.98)	0.066	1.45 (0.56–3.80)	0.444
D-dimer, 1 μg/mL increase	–	1.19 (0.96–1.47)	0.110	1.26 (0.98–1.62)	0.077

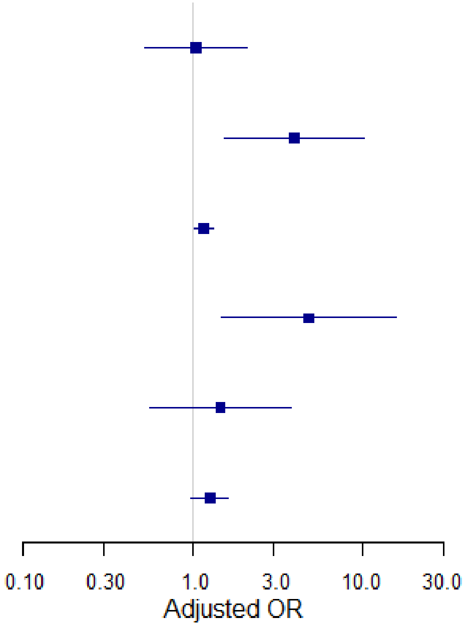

BI, Barthel Index; OR, odds ratio

Figure S2. Associations between the demographic and clinical characteristics at admission and a greater risk of functional decline in patients without very severe functional impairment before coronavirus disease 2019 onset

Characteristics	Reference	Unadjusted OR (95% CI)	P value	Adjusted OR (95% CI)	P value
Age, ≥80 years	<80 years	5.46 (2.43–12.26)	<0.001	1.54 (0.46–5.17)	0.484
Female	Male	3.83 (1.80–8.13)	0.001	3.59 (1.30–9.90)	0.014
BI, <100	100	58.84 (7.67–451.53)	<0.001	41.66 (4.50–385.33)	0.001
Dementia	Absence	10.83 (4.06–28.88)	<0.001	2.94 (0.76–11.43)	0.119
Albumin, <3.3 mg/dL	≥3.3 mg/dL	1.64 (0.78–3.44)	0.198	1.69 (0.58–4.90)	0.337
D-dimer, ≥1.0 μg/mL	<1.0 μg/mL	2.61 (1.22–5.59)	0.015	4.11 (1.28–13.23)	0.018

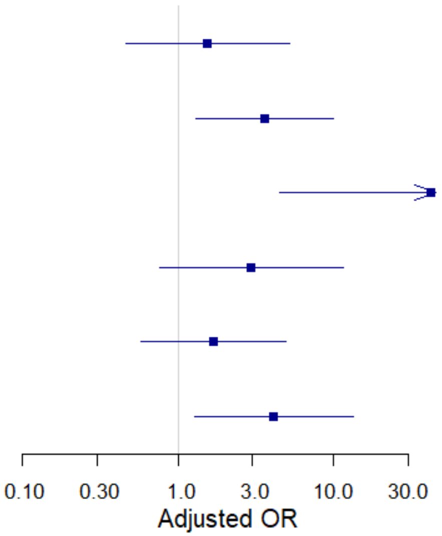

BI, Barthel Index; OR, odds ratio

Figure S3. Associations between the demographic and clinical characteristics at admission and a greater risk of functional decline or death in patients, including survivors and those who died

Characteristics	Reference	Unadjusted OR (95% CI)	P value	Adjusted OR (95% CI)	P value
Age, ≥80 years	<80 years	5.15 (2.49–10.65)	<0.001	1.91 (0.70–5.20)	0.204
Female	Male	2.87 (1.46–5.64)	0.003	2.29 (0.97–5.42)	0.060
BI, <100	100	26.13 (7.63–89.41)	<0.001	15.20 (3.84–60.22)	<0.001
Dementia	Absence	9.33 (3.99–21.82)	<0.001	1.93 (0.60–6.17)	0.268
Albumin, <3.3 mg/dL	≥3.3 mg/dL	1.50 (0.77–2.93)	0.245	1.12 (0.46–2.74)	0.803
D-dimer, ≥1.0 μg/mL	<1.0 μg/mL	2.79 (1.39–5.58)	0.005	3.36 (1.29–8.73)	0.013

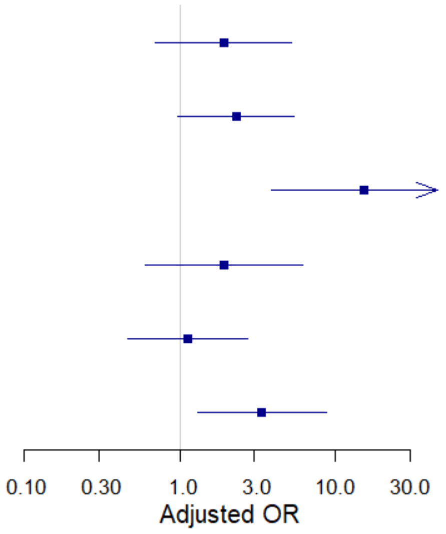

BI, Barthel Index; OR, odds ratio
